# Comparative phylogenomic analyses and co-expression gene network reveal insights in flowering time and aborted meiosis in woody bamboo, *Bambusa oldhamii* ‘Xia Zao’ ZSX

**DOI:** 10.3389/fpls.2022.1023240

**Published:** 2022-11-09

**Authors:** Wanqi Zhao, Chunce Guo, Wenjing Yao, Li Zhang, Yulong Ding, Zhenzhen Yang, Shuyan Lin

**Affiliations:** ^1^ Co-Innovation Center for Sustainable Forestry in Southern China, Bamboo Research Institute, College of Biology and Environment, Nanjing Forestry University, Nanjing, China; ^2^ Jiangxi Provincial Key Laboratory for Bamboo Germplasm Resources and Utilization, Forestry College, Jiangxi Agricultural University, Nanchang, China; ^3^ Shanghai Institute for Advanced Immunochemical Studies (SIAIS), ShanghaiTech University, Shanghai, China

**Keywords:** floral transcriptome, pseudogenization, embryo abortion, photoperiod pathway, *Bambusa oldhamii* ‘Xia Zao’ ZSX

## Abstract

Woody bamboos have peculiar flowering characteristics with intervals ranging from several years to more than 100 years. Elucidating flowering time and reproductive development in bamboo could be beneficial for both humans and wildlife. To identity the mechanisms responsible for flowering time and embryo abortion in *Bambusa oldhamii* ‘Xia Zao’ ZSX, a transcriptome sequencing project was initiated to characterize the genes involved in developing flowers in this bamboo species. Morphological studies showed that pollen abortion in this bamboo species was mainly caused by a delay in tapetum degradation and abnormal meiotic process. Differential expression (DE) and optimized hierarchical clustering analyses identified three of nine gene expression clusters with decreasing expression at the meiosis of flowering stages. Together with enriched Gene Ontology Biological Process terms for meiosis, this suggests that their expression pattern may be associated with aborted meiosis in *B. oldhamii* ‘Xia Zao’. Moreover, our large-scale phylogenomic analyses comparing meiosis-related transcripts of *B. oldhamii* ‘Xia Zao’ with well annotated genes in 22 representative angiosperms and sequence evolution analyses reveal two core meiotic genes *NO EXINE FORMATION 1* (*NFE1*) and *PMS1* with nonsense mutations in their coding regions, likely providing another line of evidence supporting embryo abortion in *B. oldhamii* ‘Xia Zao’. Similar analyses, however, reveal conserved sequence evolution in flowering pathways such as *LEAFY* (*LFY*) and *FLOWERING LOCUS T* (*FT*). Seventeen orthogroups associated with flowering were identified by DE analyses between nonflowering and flowering culm buds. Six regulators found primarily in several connected network nodes of the photoperiod pathway were confirmed by mapping to the flowering time network in rice, such as *Heading date* (*Hd3a*) and *Rice FT-like 1* (*RFT1*) which integrate upstream signaling into the downstream effectors. This suggests the existence of an intact photoperiod pathway is likely the key regulators that switch on/off flowering in *B. oldhamii* ‘Xia Zao’.

## Introduction

The transition from vegetative growth to reproductive growth is an important developmental phase in the life cycle of plants. Floral initiation is commonly regulated by various environmental and physiological cues. Bamboos (*Bambusoideae*) are a lineage of perennial forest grasses native to all continents except Europe and Antarctica. *Bambusoideae* are classified into 116 genera and approximately 1439 species (Bamboo Phylogeny Group, 2012; Clark et al., 2015; Lin et al., 2018). Bamboos consist of woody and herbaceous species. Woody bamboo species are characterized with lignified culms, complex branching, bisexual flowers, and a prolonged vegetative phase lasting from several to more than a hundred years before flowering (McClure, 1993). To biologists, the most captivating phenomenon is the extended synchronous flowering of woody bamboos which is related to the long blooming cycle (Janzen, 1976). Most woody bamboos blossom only once in their whole lifetime and usually die after flowering. Bamboo flowering is unpredictable, long-periodic, gregarious, and uncontrollable, making it unwieldy and devastating to bamboo forest resource and farmers. Once bamboos flower, the subsequent die-off can result in sudden ecological consequences-ecological environment of the bamboo forests is changed, and the economic interests of bamboo farmers and the food and living environment of pandas are no longer guaranteed. However, until now the underlying mechanism of bamboo flowering remains unclear. Therefore, it is important to ascertain the unique flowering pathways and genes involved in bamboo flowering time and floral development.

Flowering depends on many factors, including environment, nutrition, climate, and physiological status. Several hypotheses have been proposed for the flowering mechanisms in bamboo (Gielis et al., 1999; Franklin, 2004). Some MADS-box genes have been found to affect the flowering time in angiosperms (Wysocki et al., 2016). For example, *SOC1* gene in *Arabidopsis* and its homolog *OsMADS56* in *Oryza sativa* (rice) induce floral development (Ryu et al., 2009; Lee and Lee, 2010). And as antagonists of *SOC1* gene, *SVP* in *Arabidopsis* and *OsMADS22/OsMADS55* in rice control flowering (Hartmann et al., 2000; Lee et al., 2012). In certain bamboos species, several important flowering-related genes have been identified (Tian et al., 2005; Tian et al., 2006; Lin et al., 2009; Lin et al., 2010; Xu et al., 2010; Hisamoto and Kobayashi, 2013), and environmental factors and different hormones induce flowering *in vitro* (Nadgauda et al., 1990). Moreover, the gene expression studies provide insights into the mechanisms for bamboo floral initiation and development. In 2013, the first draft of nuclear genome for *Phyllostachys edulis* was published, which provides a reference genome assembly for comparisons with other bamboo species (Peng et al., 2013). Next generation sequencing with RNA-Seq method (Wang et al., 2009) has allowed gene expression to be examined at a large scale for non-model organisms. This method was first used by Zhang et al. (2012) to study the floral transcriptome in *Dendrocalamus latiflorus* (a bamboo species), and then a following study on different tissues such as leaf, stem, shoot, and root of the same species by Liu et al. (2012). Peng et al. (2013) presented transcriptome results from six different heights of moso bamboo fast growing shoots using the Illumina HiSeqTM 2000 platform. An active *Dof*-*Hd3a*-MADS-flowering pathway in moso bamboo was conducted by Gao et al. (2014). In Gao et al. (2015), suggested that a large number of differentially expressed microRNAs and their targets participated in diverse primary biological pathways and played significant regulatory roles in flowering of moso bamboo. In Ge et al. (2017), proposed that *PheDof1*, *PheMADS14* and six miRNAs may play vital regulatory roles in flower development and floral transition in moso bamboo.

In Hou et al. (2021), identified a *SOC1*-like gene, *BoMADS50*, from *Bambusa oldhamii*, whose conserved and specific SNP function may be responsible for the existence of synchronous and sporadic flowering in bamboo. Nevertheless, until now few studies have reported the anatomy of bamboo flowers and embryo development due to so long phase of vegetative growth. Meanwhile, most bamboo species have relatively low maturing rate (Zhou, 1998; Lin et al., 2018), thus only a few studies have explored the mechanism of low seed production in bamboos (Hu et al., 1994; Huang et al., 1999; Wang et al., 2006; Lin et al., 2009; Lin and Ding, 2012; Lin and Ding, 2013; Lin et al., 2015; Wang et al., 2015). *B. oldhamii* ‘Xia Zao’ is an edible shoot and woody species, with great ecological and economic values. It is preferentially distributed in subtropical regions such as Fujian province, China. Current research of *B. oldhamii* are mainly focused on plant physiology, morphology, ecology and material science. However, there have been few molecular studies on flowering of this species (Lin et al., 2010). In 2013, *B. oldhamii* ‘Xia Zao’ flowered in the Xiapu bamboo garden, China, and there are no reports that the seeds or fruits have ever been found after flowering.

In this present study, in order to investigate the mechanism of flowering time and pollen abortion in *B. oldhamii* ‘Xia Zao’, we performed a comprehensive transcriptome sequencing for gene expression profiling and explored the cytological meiotic process with paraffin section technique. Here, we collected 27 flower and nonflower samples from *B. oldhamii* ‘Xia Zao’ representing different developmental phases: culm bud, underground shoot, above-ground shoot, and spikelet with different development phases. Using a large-scale transcriptome profiling, we interrogated the expressed transcriptomes including flower tissues at different stages. These transcriptomes were analyzed for two complementary objectives: (1) identify the key regulators that switch on/off flowering in *B. oldhamii* ‘Xia Zao’ through phylogenomic and comparative sequence analyses using transcriptomic data; (2) identify the key causal floral-related genes responsible for meiotic failure by examining the abnormal microstructure. By using a *de novo* transcriptome assembly, we generated a rich resource of sequence information including a list of flower and meiotic genes. Our cytological studies revealed abnormalities associated with meiotic failure, which is likely linked to nonfunctional sequence evolution of two key genes *PMS1* and *NEF1* with known roles in pollen and tapetum development (Ariizumi et al., 2004; Li et al., 2009). Moreover, we identified the retention of conserved photoperiod pathway in *B. oldhamii* ‘Xia Zao’ that may contribute to bamboo flowering.

## Materials and methods

### Tissues, libraries, and sequence data

**Figure 1:**
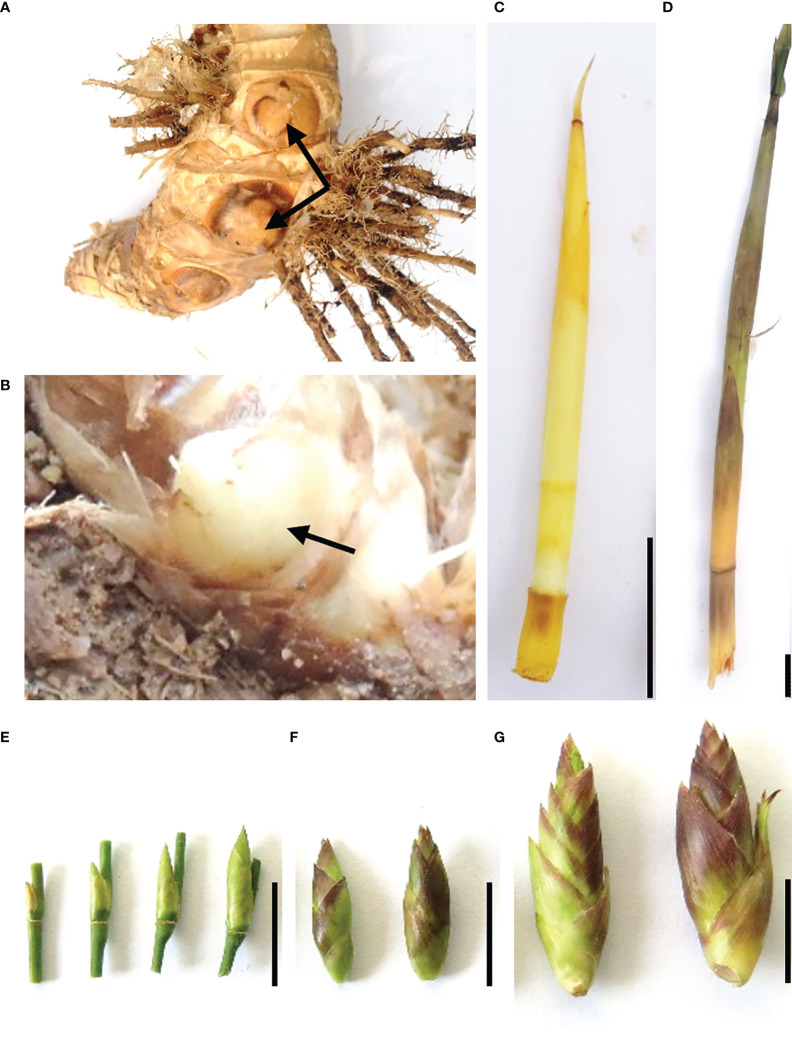
*B. oldhamii* ‘Xia Zao’ samples collected for the transcriptome. **(A)** culm buds from non-flowering plants (NF3_Cb). **(B)** Culm buds from flowering plants (F1_Cb). **(C)** Underground shoot buds from flowering plants (F1_ShU). **(D)** Above-ground shoot buds from flowering plants (F1_ShA). **(E)** Spikelet length <1 cm (F1_Fl1). **(F)** Spikelet length between 1 and 1.5 cm (F1_Fl2). **(G)** Spikelet length > 1.5 cm) (F1_ Fl3). Bar = 1 cm.

The samples of *B. oldhamii* ‘Xia Zao’ were collected on November 26, 2013 from the Xiapu breeding base, Ningde city, Fujian province (119°59′14″E, 26°48′10″N), China. A total number of 27 tissues were collected from three clumps: two flowering clumps (clump 1 and clump 2) and one non-flowering clump (clump 3). As *B. oldhamii* ‘Xia Zao’ has a lifestyle dominated by clonal reproduction, these three clumps were developed from the same asexual clone and were genetically identical. Six tissues including culm buds (here defined as F1_Cb, **Figure 1**), underground shoot buds (here defined as F1_ShU, **Figure 1**), above-ground shoot buds (here defined as F1_ShA, **Figure 1**), stage-1 flowers (spikelet length < 1cm) (here defined as F1_Fl1, **Figure 1**), stage-2 flowers (spikelet length between 1 and 1.5 cm) (here defined as F1_Fl2, **Figure 1**), and stage-3 flowers (spikelet length > 1.5 cm) (here defined as F1_Fl3, **Figure 1**) were collected from flowering clump 1. The buds of nonflowering culm base from clump 3 were collected as a control (here defined as NF3_Cb, **Figure 1**). Three biological replicates were included for above each tissue. In addition, to get an idea of the variation within different clumps, three flowering tissues of clump 2 with 2 biological replicates from each were generated (here defined as F2_Fl1, F2_Fl2, F2_Fl3). An illustration of the samples is shown in **Figure 1**. The samples were immediately frozen in liquid nitrogen and stored at -80°C. RNA extraction, library preparation, and Illumina paired-end sequencing were performed to generate RNA-Seq data with the read length of 2×100 bp and an insert size from 200 bp to 350 bp on the Hiseq 2000 platform. Library preparation and Illumina paired-end sequence followed the procedure described in Illumina Truseq™ RNA sample prep kit.

### Assembly, cleaning, and annotation

A total of more than 1.5 billion Illumina paired-end RNA-Seq reads were generated from 27 bamboo cDNA libraries (**Table 1**). To decrease the amount of data for further processing, duplicate reads were removed with Prinseq (Schmieder and Edwards, 2011). Adapter sequences were detected using fastQC and Trimmomatic (Bolger et al., 2014) was used to remove adapter sequences and low-quality bases. A *de novo* transcriptome assembly was subsequently performed with Trinity release (Grabherr et al., 2011) using cleaned reads from all the 27 tissues. The resulting assembly was further cleaned by removing contigs without coding regions (Iseli et al., 1999) and redundant transcripts using the Assembly Post Processing pipeline in PlantTribes (https://github.com/dePamphilis/PlantTribes). Further, a representative sequence was selected to reduce the complexity of the data for downstream analyses. The procedure to select representatives from the transcriptome followed the procedure described by Yang et al. (2015). Contigs were classified into 22-genome orthogroup classification using HMMScan, and one longest sequence was selected from a set of unigenes that fall within the same Trinity component and the same orthogroup. A final cleaned transcriptome assembly was generated to contain 167,058 representative sequences that retain sequences with open reading frames (ORF) greater than 200 bp. The coding sequences (CDS) and peptide sequences (PEP) were predicted using ESTScan. Sequences were further classified into the 22-genome orthogroup classification (Yang et al., 2015) using the Gene Family Classifier pipeline in PlantTribes (https://github.com/dePamphilis/PlantTribes). The putative function of each sequence was analyzed with InterproScan, Pfam analyses, and annotation information (including the best *Arabidopsis* hit, GO information) from its assigned orthogroup.

**Table 1:** Trinity assembly statistics for the combine transcriptome of the 27 samples of *B. oldhamii* ‘Xia Zao’.

Category	Trinity
Number of Illumina raw sequence reads	1,531,306,412
Number of filtered Illumina reads	1,472,785,204
Number of representative contigs	167,058
Number of representative contigs assigned to an orthogroup	78,568
N25 (bp)	1,422
N50 (bp)	657
Average assigned representative length (bp)	1,056
Min. assigned representative contig length (bp)	201
Max. assigned representative contig length (bp)	16,267

### Read mapping and quantification

Filtered clean reads from each library were mapped onto the final cleaned assembly using bowtie2 (Langmead and Salzberg, 2012) with default parameters. A gff file with the length of each transcript was generated, combined with the mapping output BAM file, expression quantification was performed with featureCounts (in SubREAD (Liao et al., 2014). Reads were combined by including paired-end reads and single reads using featureCounts (Liao et al., 2014). A gene expression file was generated to contain read counts for each gene from each library. The gene expression file was then filtered to remove contigs with mean read count across all the libraries less than 1. Read counts mapped in each library were then normalized by fragments by kilobase per million mapped reads (FPKM) (Mortazavi et al., 2008) that accounts for bias in contig length as well as the sequencing depth from each library.

### Sample clustering with pvclust and principal component analyses

Problematic libraries identified using pvclust (Suzuki and Shimodaira, 2006) with the assumption that replicates within one sample should group with each other. Bootstrapped sampling clustering was performed by the pvclust (Suzuki and Shimodaira, 2006) command in R using the gene expression matrix that quantifies the expression of each gene in log2FPKM across each library. Correlation distance and complete clustering were adopted. Replicated libraries that didn’t group with the other replicates within the same sample were discarded. PCA was performed with the rlog transformed read counts by rlogTransofrmation in DESeq2 and prcomp function in R. The code for PCA analyses is also available on GitHub.

### Identification of differentially expressed genes

DE analyses were performed between any two samples using DESeq2 (Love et al., 2014) where the rlog transformation (rlogTransofrmation function in R) significantly increases the power of DE identification. Read counts of all genes from each two tissues were used for DE analysis. A P-value cutoff of 0.01 and a fold-change cutoff of 2 were required for DE genes.

### Clustering analyses

We utilized an optimized hierarchical clustering (Bar-Joseph et al., 2001) using a customized R function for clustering analyses. The optimized hierarchical clustering is preferred as it adopts an improved measurement of gene-and-gene distance – correlation distance, instead of Euclidean distance (Bar-Joseph et al., 2001). The advantage is that it identifies more tightly linked cluster of genes with similar expression pattern across all stages regardless of their absolute level of expression. The gene expression matrix in log2FPKM was used as the input of hierarchical clustering. Then the top 4000 genes with the median absolute deviation (mad function in R) were used for optimized hierarchical clustering. Overall gene expression clustering was then examined, and the number of clusters was selected based on the transcriptional pattern. Each cluster of interest was identified by applying function “hclust” and “cuttree” function.

### Identification of known floral and meiosis genes

Using a comprehensive literature search from the published functional characterization studies on flowering process in *Arabidopsis* and rice, we curated a list of genes involved in floral development regulatory network (**Supplementary Table S3**), as well as in meiosis (**Supplementary Table S2**). We also expanded the list of meiosis genes by adding a set of genes with tissue specific expression in pollen mother cells from an RNA-Seq study by Tang et al. (2010), which we term as PMC genes.

### Identification of unannotated genes in *Phyllostachys*

Several orthogroups miss *Phyllostachys* genes in the annotated gene models, such as orthogroup 7224 (*LEAFY*) (Weigel et al., 1992), orthogroup 2086 (*FT*), the absence of which is likely due to poor annotation. To recover the unannotated genes in *Phyllostachys*, we performed a TBLASTN search against the *Phyllostachys* genomic scaffolds using the predicted peptide sequences either in *B. oldhamii* ‘Xia Zao’ or rice. We then identified the genomic hit in *Phyllostachys* (Peng et al., 2013) (by creating a gff file from the aligned boundary and used “fastaFromBed” function to get the genomic sequence), which was then aligned with the *B. oldhamii* ‘Xia Zao’ query CDS sequences to identify possible exon-intron boundaries. Alternatively, if this method failed to identify clear boundary and if the gene of interest is quite conserved, we used the full-length CDS sequence of rice to identify the genomic hits in *Phyllostachys*. Then the exonic regions of *Phyllostachys* sequences were used to predict possible peptide sequences with all possible six frames; the correct frame was determined as the one that well aligned with the predicted peptide sequences in *B. oldhamii* ‘Xia Zao’ or *Oryza*.

### Sample fixation, paraffin section preparation and sample staining

Sample fixation and preparation followed the procedure described by Li (1991). Samples were taken and directly fixed in formaldehyde acetic acid (FAA: 35-40% formaldehyde, 70% alcohol and 100% acetic acid in a proportion of 5:90:5 v/v) at 4°C until preparation. At the start of preparation, the samples were treated with Ehrfich’s haematoxylin as a whole, then washed in running water. After the samples became blue, 50%, 70%, 85%, 95% and 100% ethanol were used successively (2×1.5-2 h) and then kept for 1h in solution of xylol and ethanol in proportions 1:1, next treated with pure xylol for 1 h (2×), and then kept 2-3 h in solution of xylol and paraffin in proportions 1:3, 1:1 and 3:1 successively, placed in a mixture of xylol-paraffin and kept at 58°C, stored overnight allowing xylol to evaporate. Then the samples were kept in pure paraffin for 2-3 h (3×). Finally, the samples were embedded in paraffin. Transverse sections (7μm) were cut using a rotary microtome (Leica RM2255) and double stained with 1% alcoholic Safranin O (Sigma S-2255) (in 50% ethanol) and 1% Fast green (Fluka 05500). The sections were permanently mounted in Canada balsam. Throughout the observations, images were captured with a microscope (Leica DM5000B). At least 3 replications were caried out for each experiment and micrograph.

### RNA extraction, cDNA synthesis and reverse transcription quantitative real-time PCR

Total RNA was extracted from the collected samples according to the manufacturer’s instructions (RNA pure Ultra-pure Total RNA Rapid Extraction Kit, Yuanpinghao Bio). The purity/concentration of RNA samples was determined using a NanoDrop 2000c spectrophotometer (NanoDrop, Thermo Scientific). Samples with concentrations greater than 100 ng.ml^-1^ and an optical density absorption ratio A260/A280 greater than 1.8 were used for cDNA synthesis and the integrity of RNA bands were detected by 1.0% agarose gel electrophoresis. Individual RNA samples were stored at -70°C and then 1 μg of total RNA were used as template in RT reactions with the PrimeScript RTase reverse transcriptase (TaKaRa), according to the manufacturer’s instructions.

The primers for 12 genes from *B. oldhamii* ‘Xia Zao’ were designed by using the Primer 5.0 software, referring to the **Supplementary Table S5**. (http://www.genome.wi.mit.edu/cgi-bin/primer/primer5.cgi). All primer pairs were initially tested *via* standard RT-PCR using the Premix Ex Taq (TaKaRa) and the presence of a single amplification product of the expected size for each gene was verified by electrophoresis on a 2.5% agarose gel. cDNA products for qPCR were diluted 10-fold prior to use in real-time PCR. To ensure the absence of contamination or primer-dimer formation for each primer pair, a template-free control reaction was run. qRT-PCR reactions were carried out in 96-well blocks with an Applied Biosystems StepOne Plus Real-Time PCR system using TransStart Tip Green qPCR SuperMix (TransGen Bio). The reaction conditions were recommended by the manufacturer (30 s at 95°C, 40 cycles of 95°C for 5 s, and 55-60°C for 30 s). The dissociation curve was obtained by heating the amplicon from 60 to 95°C. All qRT-PCR reactions were carried out in triplicates. Each reaction was repeated three times, and the expression levels were calculated using the 2^−ΔCT^ method (Livak and Schmittgen, 2001). Finally, RNA-seq-derived expression levels were compared with those derived from qRT-PCR.

## Results

### Microspore and male gametophyte development and anther cytological abortion of *B. oldhamii* ‘Xia Zao’

**Figure 2:**
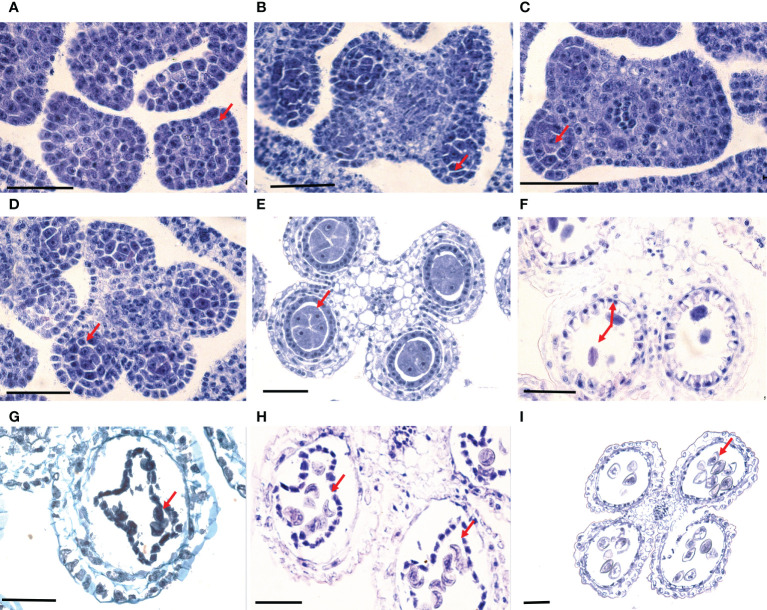
Normal development of microsporocytes yet anther abortion of *B. oldhamii* ‘Xia Zao’. **(A)** Undifferentiated anther. **(B)** Archesporial cell divided periclinally to produce one primary parietal cell and one primary sporogenous cell. **(C)** Primary sporogenous cell and anther wall with two layers. **(D)** Secondary sporogenous cells and anther wall with three layers. **(E)** Microspore mother cells and anther wall with four layers. **(F)** Diad, showing more dense cytoplasm, abnormal spindle fiber and unformed equatorial plate. **(G)** Deformed tapetum and abnormal tetrad. **(H)** Most uninucleate microspores shrank and deformed abnormally. **(I)** Deformed 2-cell pollen grains and the residual tapetum. Bar = 50 μm.

Each floret of *B. oldhamii* ‘Xia Zao’ has six anthers, and each anther has 4 sporangiates. At the stage when the spikelet length was less than 0.5 cm, the anther was still in an undifferentiated state, a bulky differentiated sporogonium can be seen beneath the epidermis cells at the corner of the anther on the transverse section (**Figure 2**). Porogonium divides periclinally to produce a primary parietal cell and a primary sporogenous cell (**Figure 2**). During the early developmental stage of secondary sporogenous cells, there were only a few secondary sporogenous cells identified in loculus (**Figure 2**). The young anther walls usually consisted of three layers: from the outer to the inner layers, epidermis, endothecium and secondary parietal cell layer (**Figure 2**), at this time the spikelet length was < 1.0 cm (stage FI1). The structure of anther wall was fully developed in the microsporocyte stage. Outside-in layers of the anther wall, the epidermis, endothecium, middle layer and tapetum, can be seen in specimen (**Figure 2**). At this point, the length of spikelet grows to 1.5 cm (stage FI2). When the spikelet length was > 1.5 cm (stage FI3), microsporocytes would go into a period of meiosis. The abnormal meiotic behaviors such as a denser cytoplasm, abnormal spindle fiber and unformed equatorial plate were frequently observed (**Figure 2**). At the stage of microspore development, degeneration was still observed in the anther such as unclear nucleus in pollen grains and distorted tapetum (**Figure 2**). At the vacuolated uninucleate microspore stage, some microspores shrank seriously, resulting in deformation and degeneration of these microspores (**Figure 2**). Even when the 2-cell pollen grains were formed, their pollen walls were shrunk and aberrant, and the residual tapetum could still be observed (**Figure 2**). In some anthers, almost all microspores deformed, and no uninucleate microspores could successfully develop into pollen grains.

### Sample collection and assembly statistics

More than 1.5 billion reads were generated from six major developing tissues during bamboo floral development (**Figure 1**) and resulted in 942051 contigs in the Trinity assembly. These tissues include culm base buds from flowering (F1_Cb) and nonflowering plants (NF3_Cb), underground (F1_ShU) and above-ground shoot buds (F1_ShA), and florets of different size (F1_Fl1, F1_Fl2, F1_Fl3) representing different stages of flower development. After filtering and selecting representatives (following Method “Assembly, cleaning, and annotation”), 167,058 contigs with an average unigene length of 766 bp and an N50 unigene length of 380 bp were represented in the final cleaned non-redundant assembly (**Table 1**). To examine if shorter contigs were attributed to possibly artificial unigenes constructed from traversing the *de brujin* graph path in Trinity assembly, we only considered 78,568 contigs that are assigned to an orthogroup (**Table 1**). The N50 statistics showed a two-fold increase of 657 bp, and the average sequence length was 1056 bp (**Table 1**). To evaluate the completeness of the transcriptome assembly, we examined the capture rate of the ultra-conserved orthologs (UCOs) gene set. The gene capture rate of 99.1% (**Table 2**) indicates a fairly complete transcriptome assembly.

**Table 2:** Transcriptome gene capture of ultraconserved single copy genes.

UCOs genes	UCO orthologs in the transcriptome	Proportion
357	354	99.1%

### Global transcriptional profile

**Figure 3:**
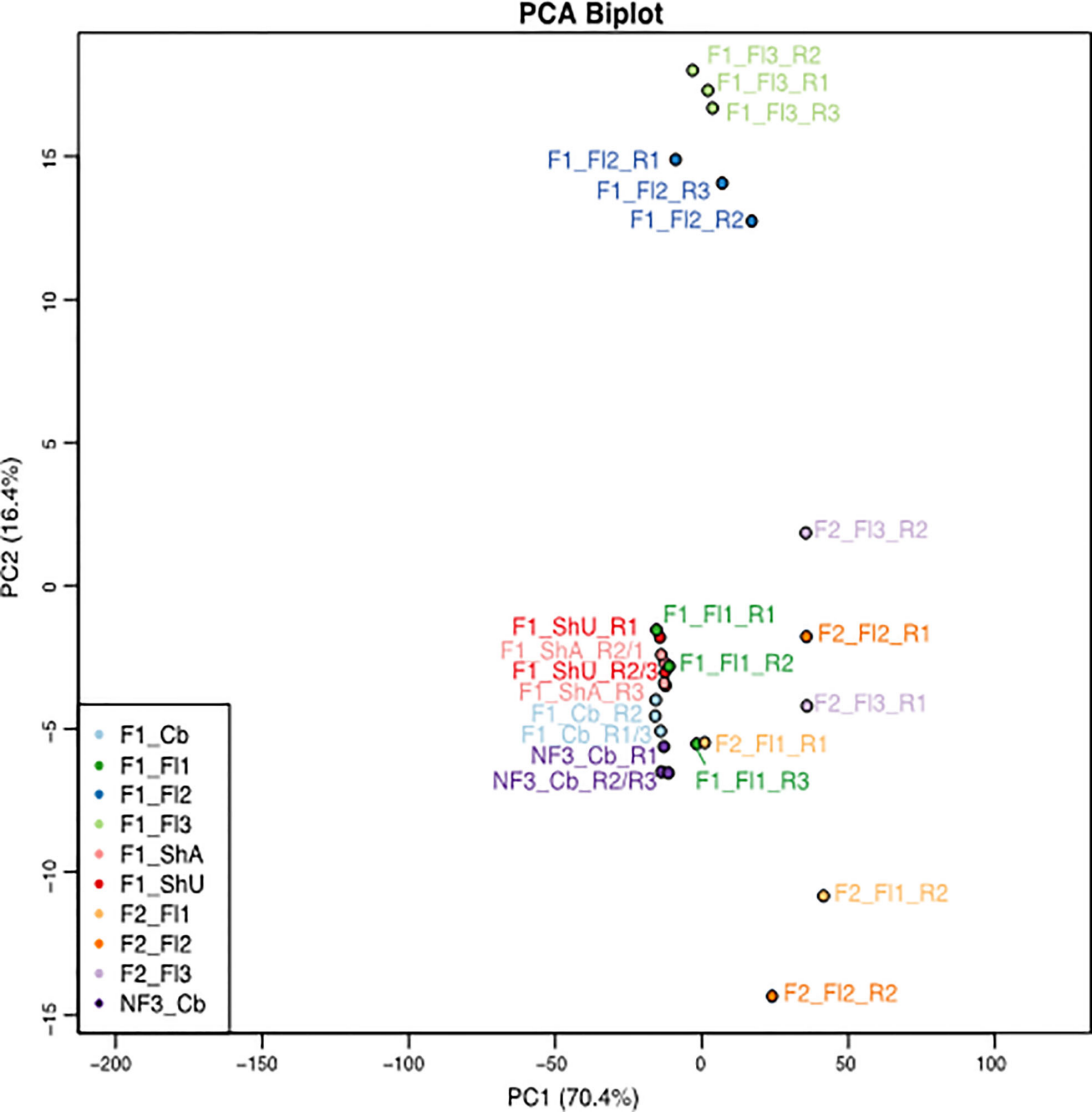
PCA plot of the 27 samples of *B. oldhamii* ‘Xia Zao’ generated for this transcriptome study. The samples include one culm buds tissue of three biological replicates from nonflowering clump 3 — labeled as “NF3_Cb”, six tissues each with three biological replicates from flowering clump 1 – labeled as “F1”, as well as three tissues each with two biological replicates from flowering clump 2 – labeled as “F2”. “Cb” – culm buds, “ShU” – underground shoots, “ShA” – aboveground shoots, “Fl1” – flower stage 1 with each spikelet length less than 1 cm, “Fl2” – flower stage 2 with each spikelet length between 1cm and 1.5 cm, “Fl3” – flower stage 3 with each spikelet length greater than 1.5 cm. spikelet.

Three problematic libraries, F1_ShU_R1, F1_Fl2_R2, and F2_Fl1_R1 (**Figure 3**) were identified by Pv-clustering analyses (Suzuki and Shimodaira, 2006). Because they are not grouped with the other replicate libraries of the same tissue, it represented some inconsistency within-sample replicates that could be due to sample harvest, RNA extraction, or sequencing process, and was valid for removal.

The global transcriptional profile by the PCA plot reveals that the biggest variation between the samples lies between clump 1/3 and clump 2 tissues, as the first principal component separating clump 1 (together with clump 3 culm buds) and clump 2 floral tissues explains 70.4% of the variation (**Supplementary Figure S1**). The second principal component shows that floral stage 2 and floral stage 3 are most distinct from the remaining vegetative tissues and floral stage 1 tissues from clump 1 and nonflowering clump 1 culm buds. Interestingly, culm buds’ tissue from flowering clump 1 and nonflowering clump 3 are more similar to each other, indicating a similar transcriptional profile within the same culm buds tissue despite from different clones (**Figure 3** and **Supplementary Figure S1**). The unexpected grouping of F1_Fl1_R3 and F2_Fl1_R1 (**Figure 3**) again support these two samples are possible outliers. The three replicates of flower 2 (blue) and flower 3 (light green) in clump 1 show tight correlation. Conversely, the clump 2 flower tissues show greater within-sample correlation than between sample variation. In addition, there is a higher between-clone variation in reproductive tissues (between clump 1 and clump 2) than in vegetative culm buds’ tissues (between clump 1 and clump 3). Because of this high level of variation from clump 2, samples from clump 1 (6 tissues that start with “F1”) and clump 3 (one tissue that start with “NF”) were focused for downstream differential expression and clustering analyses in flowering evolution of *B. oldhamii* ‘Xia Zao’.

### Validation of digital gene expression by qRT-PCR

**Figure 4:**
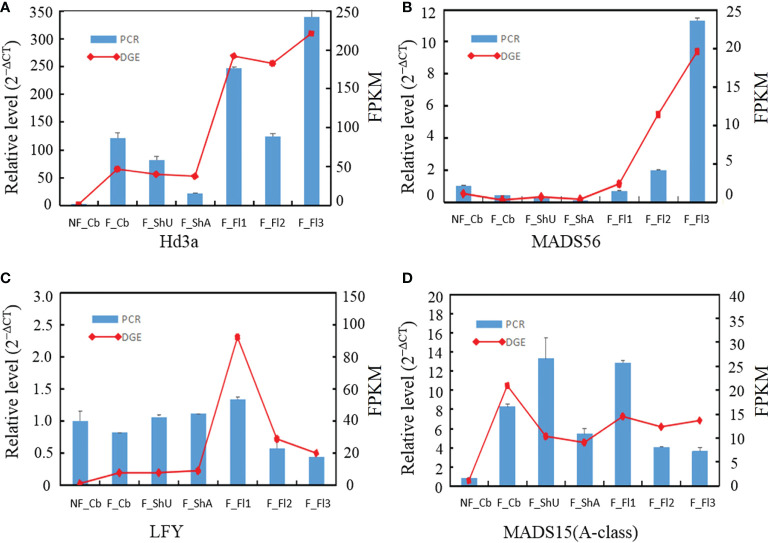
qRT-PCR validation of the expression for four selected genes in different developmental stages. The transcript levels were normalized to that of PP2A and UBC18.

To confirm the genes in different development samplings, ten genes were randomly selected for qRT-PCR. Expression of four genes (*Hd3a*, *MADS56*, *E class* gene, *MADS15*) by qRT-PCR was fitted well with the pattern of Tag-seq analysis (**Figures 4A–D**). And another eight genes (*B class*, *C class*, *E class*, *ZIP4*, *TDR*, *PTC1*, *CYP703A3*, *G class*) similarly behaved between qRT-PCR and deep-sequencing methods (**Supplementary Figure S2**).

### Differential gene expression analyses

Pairwise DE analyses were performed between any two samples of the seven tissues from the nonflowering clump 3 and flowering clump 1. As a result, 21 DE analyses were generated that identified a number of DE genes between any of the seven tissues. The biggest variation is revealed almost between clump 3 and clump 1 tissues, except between the culm buds’ tissues from the two (**Supplementary Figure S3**). It suggests that the variation between clump is smaller than the variation between tissue from the same clump. Within clump 1, the variation is greatest between culm buds and stage-3 floral tissues (12,587 DE unigenes). Significant difference was also revealed between stage-1 floral tissues and stage-3 floral tissues (7712 DE genes).

### Abundant floral tissue expression accounts for the major transcriptional profile

**Figure 5:**
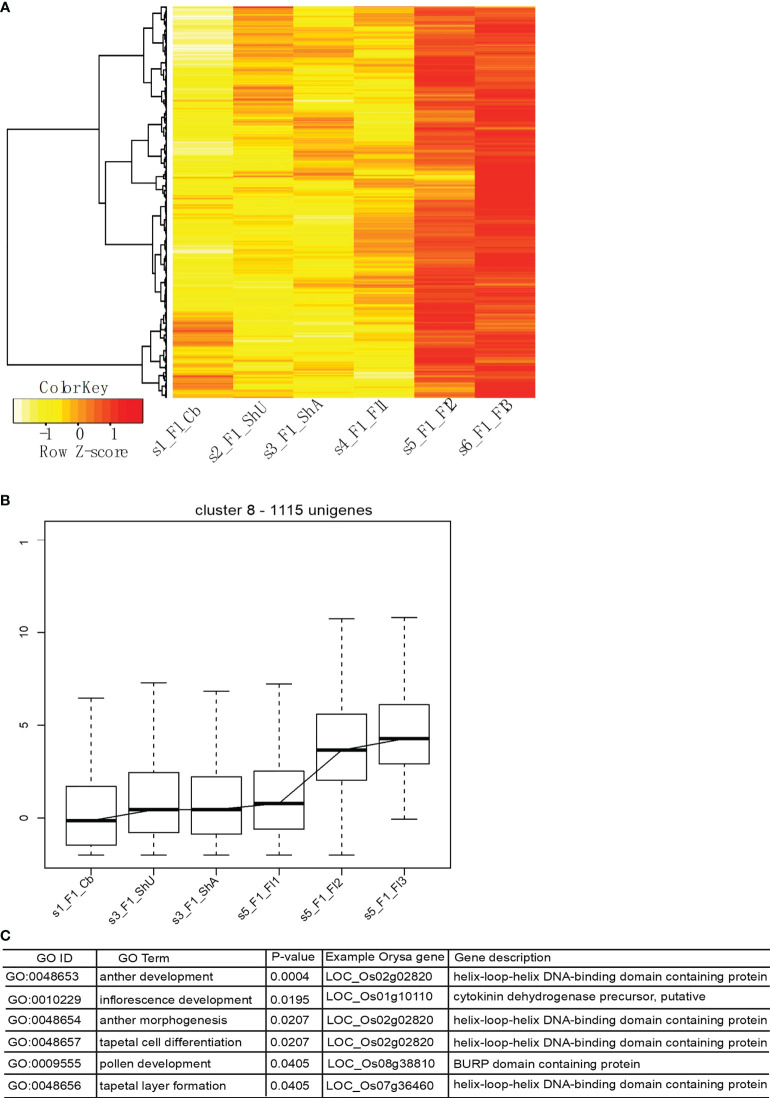
The major transcriptional profile includes genes highly expressed in flower tissues and functional enrichment shows the enriched Gene Ontology Biological Process terms. **(A, B)** show the expression profile of genes in this cluster, and **(C)** shows the enriched functional categories and representative gene in rice.

Our optimized clustering identifies a total of nine global transcriptional clusters (**Supplementary Figure S4** and **Table S1**) based on the top 4000 genes that show the highest expression deviation from the median. Cluster 1 shows a dominant signature of flower gene expression with upreuglated expression in all three flower stages, but quite low level of expression in vegetative tissues (culm buds, undeground and above ground shoot buds). Cluster 2, however, shows an opposite pattern of vegetative tissue specific expression. Cluster 3 appears to capture genes specifically expressed in shoot, likely contributed by genes involved in rapid cell expansion. Each cluster is represented by a different set of genes with the numbers vary across clusters. The major expression pattern is characterized with abundant expression in flower stage 2 and stage 3 in cluster 8 (**Figure 5A**), indicating a large number of genes contribute to the floral transcriptome.

To identify if unigenes from cluster 8 encode the genes involved in meiosis development, we did an enrichment analysis using this set of genes. Protein sequences were used to blast search against the rice peptide sequences to obtain the best hits, which were used for Gene Ontology enrichment analysis using the webtool (http://www.ricearray.org/analysis/overview.shtml). As expected, several GO Biological process (GO BP) categories related to meiotic development were significantly enriched among this set of genes, which include inflorescence development, pollen development, anther morphogenesis, anther development, tapetal cell differentiation, and tapetal layer formation. These categories were mapped onto six genes with a known role in flower regulation and development in rice, which include two genes (LOC_Os02g02820 (*AMS* in *Arabidopsis*) (Sorensen et al., 2003) and LOC_Os07g36460) encoding a helix-loop-helix DNA binding transcription factor (TF)) and one encoding a *FT-like 2* homologous to *Flowering locus T* gene (LOC_Os06g06320, *Hd3a* from orthogroup 2086) (**Figure 5**).

### Misregulated transcriptional profile likely associated with meiotic failure

As morphology of *B. oldhamii* ‘Xia Zao’ meiotic processes indicates evidences of meiotic abortion (**Figure 2**), we attempted to look for any abnormal patterns related to expression. From the nine overall transcriptional clusters (**Supplementary Figure S4**), we identified three clusters – cluster 5, cluster 7, and cluster 9 that could be associated with possible meiotic failure (**Supplementary Figure S5**). All these three clusters display high expression in at least one flower stages, yet show decreased expression towards flower 2 or flower 3 stage (**Supplementary Figures S5A–C**), indicating a failure to complete the meiotic development. However, the number of genes from these three clusters (682) accounts for only a small number of genes, especially when it comes to comparison with the 1115 unigenes from the major transcriptional cluster 8. Nevertheless, this indicates a possibility of misregulation of gene expression which could be associated with meiotic failure. Gene enrichment analysis identified a distinct set of functional enriched terms from the major cluster 8 (**Supplementary Figure S5D**), suggesting genes involved in processes such as meiosis, megasporogenesis, and tapetal layer development may be misregulated to cause possible meiotic failure, whereas genes related to enriched functional terms from cluster 8 such as pollen development don’t appear to be responsible for meiotic failure of *B. oldhamii* ‘Xia Zao’.

### Conserved *AMS* sequence evolution in *B. oldhamii* ‘Xia Zao’

**Figure 6:**
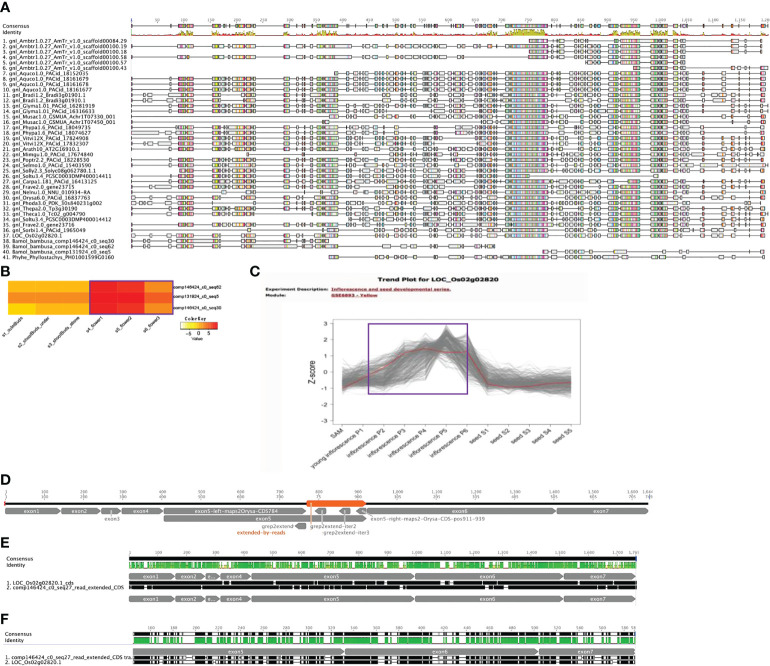
Conserved *AMS* protein sequence evolution in *B. oldhamii* ‘Xia Zao’. **(A)** Orthogroup protein multiple sequence alignment (MSA) shows fragmented *AMS* contigs in *B, oldhamii* ‘Xia Zao’. **(B)** and **(C)** show different expression trend of *AMS* gene expression in flower stages, **(B)** shows expression of *AMS* contigs in *B. oldhamii* ‘Xia Zao’, **(C)** shows expression of rice *AMS*. The purple line in B and C indicates the trend of *AMS* gene expression in *B. oldhamii* ‘Xia Zao’ and rice, respectively. **(D)** A schema illustrates the process of full-length CDS construction. Exon 1, exon 2, exon 3, exon 4, exon 6, exon 7 were constructed by high sequence similarity in assembled contigs with rice CDS. The left region of exon 5 up to 784 bp of rice *AMS* CDS and the right region of exon 5 from 911 to 939 were constructed by comparing *B. oldhamii* ‘Xia Zao’ transcriptome *assemblies to* rice CDS. The gap in the middle of exon 5 (orange region labeled as “extended-by-reads”) were filled by using read sequence data. In this process, three seeds were used to perform “grep” command in the reads to extend the right regions, and three iterations (“grep2extend”, “grep2extend-iter2”, and “grep2extend-iter3”) were able to fill the gap in exon 5. **(E, F)** are the pairwise alignment (from Geneious) of full-length *AMS* CDS and peptide sequence between *B. oldhamii* ‘Xia Zao’ and rice.

As one of the enriched flower associated functional categories from cluster 8 is anther development and *AMS* (LOC_Os02g02820 in **Figure 5**) is a key gene involved in this process (Sorensen et al., 2003), we examined if *AMS* shows any nonconserved sequence evolution in *B. oldhamii* ‘Xia Zao’. Orthogroup peptide multiple sequence alignments (MSA) shows three fragmented *AMS* contigs, two align on the N-termiinal, and one aligns on the C-terminal, and a gap between the three sequences indicate failure to assemble the full-length *AMS* (**Figure 6**). To examine whether this was due to assembly artifact, we aligned the rice *AMS* CDS with these three *B. oldhamii* ‘Xia Zao’ *AMS* contigs. By also searching aginst the original trinity assembly, and also by filling the gaps (gap mainly exists in exon 5) using raw read sequence data (**Figure 6**), we managed to construct the full-length *AMS* sequence which contains seven exons in the CDS (**Figure 6**). In the end, the full-length CDS of *AMS* encodes a quite conserved protein with a length of 547 AA. The protein sequences of *AMS* in *B. oldhamii* ‘Xia Zao’ and rice show 64.3% identity (**Figure 6** and **Supplementary Table S4**), and CDS sequences show 73% identity (**Figure 6** and **Supplementary Table S4**). The correct placement of *B. oldhamii* ‘Xia Zao’ *AMS* gene as a sister to its ortholog in *Phyllostachys* and rice indicates conserved protein sequence evolution for *AMS* gene on the sequence level (**Supplementary Figure S6A**). This indicates high level of complexity in transcriptome assembly, and for conserved genes, manual curation is possible to create the full-length coding sequences. In conclusion, *AMS* is not missing in *B. oldhamii* ‘Xia Zao’ even though evidences suggest meiosis don’t function properly in *B. oldhamii* ‘Xia Zao’ (**Figure 2**). However, downregulation of *AMS* in *B. oldhamii* ‘Xia Zao’ flower 3 stage (**Figure 6**) is different from nonreduced expression of rice *AMS* (**Figure 6**), suggesting that a noncanonical gene regulation might be associated with late stage of meiosis.

### Nonconserved sequence evolution for *PMS* gene might be associated with meiotic failure

**Figure 7:**
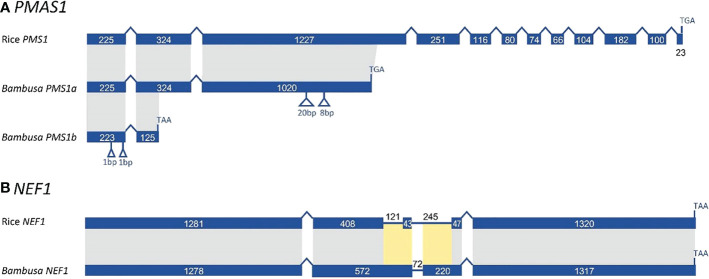
Gene structure schema of *PMS1* **(A)** and *NEF1* **(B)** gene from *B. oldhamii* ‘Xia Zao’ and rice reveals nonsense mutations causing premature stop codons. Insertion is marked byblue arrow and orange label, stop codon is indicated by a blue arrow.

In examining sequence evolution for all meiotic orthogroups, we identified an 8-bp insertion in exon 3 of *B. oldhamii* ‘Xia Zao’ *PMS1* gene (orthogroup 8661), the full-length CDS of which was constructed by comparing transcript comp149596_c0_seq1 with rice CDS. Alignment between *B. oldhamii* ‘Xia Zao’ *PMS1* transcript with rice *PMS* CDS reveals a nonspliced 695bp-intron8 in *B. oldhamii* ‘Xia Zao’ CDS (**Figure 7** and **Supplementary Figure S7**). The constructed full-length contains 12 exons in the CDS and high level of sequence similarity of 84.7% identity with rice CDS. Close examination of the two sequences reveals an 8-bp insertion in exon 3 of *PMS*, which is supported by reads, and has disrupted the frame by 2 bp and causes a premature stop codon resulting in a much truncated protein. It is believed that this small insertion may be associated with meiotic failure in *B. oldhamii* ‘Xia Zao’, as this gene plays a role in DNA mismatch repair and mutation in *Arabidopsis* shows significant decrease in seed production (Li et al., 2009), which is consistent with the reduced seed production in *B. oldhamii* ‘Xia Zao’.

### Identify floral initiation genes in early stages of *B. oldhamii* ‘Xia Zao’ development

**Figure 8:**
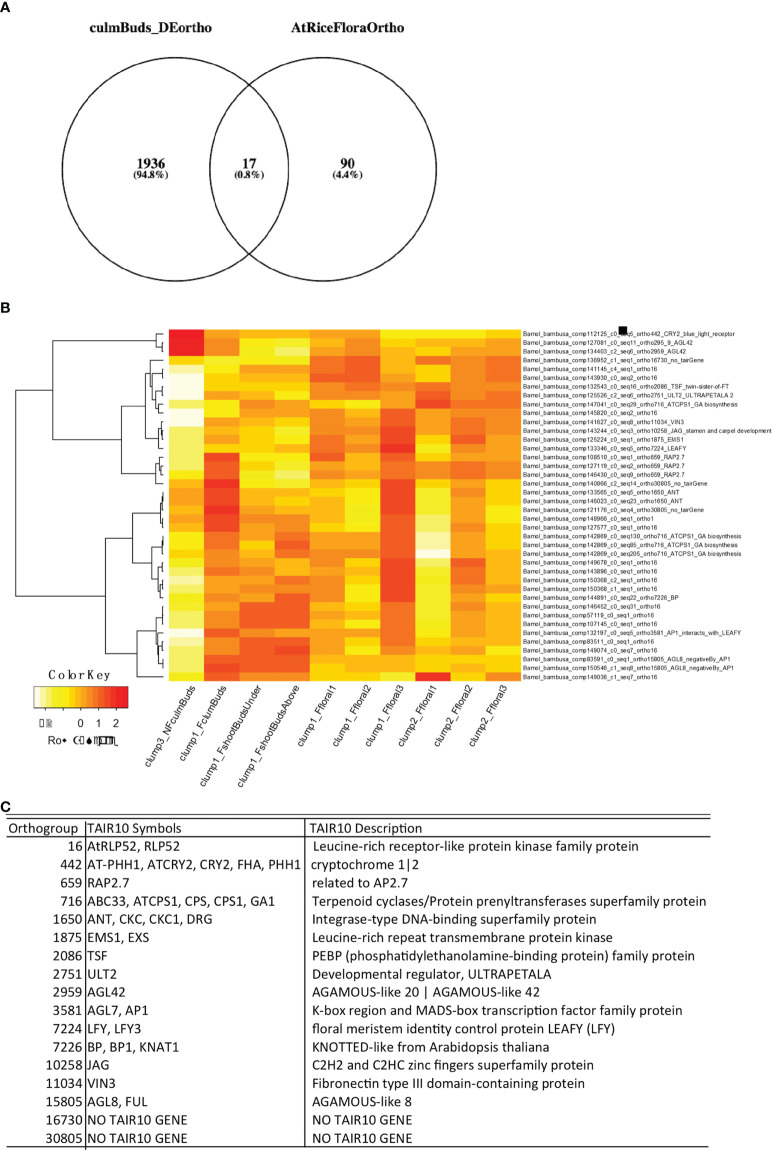
Early floral initiation genes in *B. oldhamii* ‘Xia Zao’. **(A)** Genes from 17 floral orthogroups were differentially expressed in culm buds stages in bamboo flowering tissues. **(B)** Gene expression heatmap of *B. oldhamii* ‘Xia Zao’ orthologs of known floral initiation genes curated in *Arabidopsis*. **(C)** The description of the 17 floral initiation orthogroups.

One objective of *B. oldhamii* ‘Xia Zao’ floral transcriptome is to investigate if it shares the same floral initiation pathway as other flowering plants such as *Arabidopsis* and rice. To identify if floral initiation genes were already expressed in early developmental stages such as culm buds, we performed differentially expressed (DE) analyses between nonflowering culm buds and flowering culm buds and showed that a large number of selected genes known to function in pollen developmental and meiosis were significantly enriched at early flowering stage (F_FI1) (**Supplementary Figure S8**). A total number of 3080 contigs from 1953 orthogroups defined by 22 sequenced plant genomes (**Figure 8**) were identified, which we cross-checked with our curated list of floral genes (**Supplementary Table S3**). We claim a *B. oldhamii* gene an ortholog of known floral gene when it is assigned to the same orthogroups for known floral genes from rice or *Arabidopsis*. This identified 17 known floral orthogroups from the DE transcripts between nonflowering and flowering culm buds (**Figure 8**). Only four contigs showed a reduced expression in flowering culm buds compared to nonflowering culm buds, with a majority showing an upregulated expression in flowering culm buds (**Figure 8**). These floral regulators that were expressed as early as culm buds include genes such as *LEAFY* (orthogrup 7224), *AP1* which interacts with *LEAFY* (Liljegren et al., 1999), *VIN3* (Kim and Sung, 2013) and *GA1* (Wilson et al., 1992) (**Figure 8**), a group of genes with known roles in integrating various environmental factors to regulate flowering. By using *Phyllostachys* genome and conserved sequences in rice, we identified the full-length coding sequence of *FT* (**Supplementary Table S4**), *LEAFY* (**Supplementary Table S4**), two genes with well-known roles in flower initiation. Surprisingly, instead of relaxed sequence constraint (**Supplementary Figures S6B, C** and **Table S4**), they all retain high level of sequence conservation. This suggests the retention of key processes involved in control of flowering time in at least two sequenced bamboo species, *B. oldhamii* ‘Xia Zao’ and *Ph. edulis*.

Our gene expression network analysis was based on the sequence assembled by the *de novo* transcriptome, and we didn’t really rely on for the association analysis. We mainly focused on the overall expression pattern of the gene. Additionally, we conducted a large number of comparative studies with reference to the known flowering time network of rice, and the reason we selected rice over bamboo is that the flowering pathway research mechanism in rice is relatively unambiguous. We pay attention to several important ways to regulate flowering time, such as photoperiod pathway, autonomous pathway, vernalization pathway and so on. We identified the differentially expressed genes in flowering and nonflowering tissues by blast to find their best hit in rice, and then compared these flowering pathways are conserved in bamboo. The results showed that only genes in the photoperiod pathway were found to be differently expressed in flowering and nonflowering tissues. The significance of this work is that we have demonstrated that the flowering pathway of bamboo may be complete, and the reason it is so difficult to bloom is due to genetic regulation. Our sampling and analysis also show that the photoperiod pathway may play a major role in controlling the response of bamboo to flowering or not (**Supplementary Figure S9**).

## Discussion

There are no seeds or fruits have ever been found after flowering in *B. oldhamii* ‘Xia Zao’. In this study, various stages of anther development were discerned with microscopic observation of transverse sections. There are several types of abortions observed in the development phases of anthers in *B. oldhamii* ‘Xia Zao’. For example, abnormal diad cells were observed during meiosis. Similar phenomenon was also reported in other bamboo species, such as *Menstruocalamus sichuanensis* (Lin et al., 2009) and *Shibateae chinensis* (Lin and Ding, 2012). Li et al. (2010) also reported that the cease of development led to the deformation of uninucleate pollen in one cultivars of sterile chrysanthemum. Besides, at the stage of microspore, distorted tapetum was still observed in the anther. Therefore, it could be concluded that the aberrant tapetal behaviors are associated with male sterility (Li et al., 2010; Meric et al., 2003; Shi et al., 2010). Holford et al. (1991) summarized the following three types of abnormal tapetal behavior in a male sterile onion: the premature breakdown of the tapetum at tetrad stage; the hypertrophy of the tapetum after the diad stage followed by its premature autolysis; the tapetum remaining in good condition but for an abnormal long period of time. Buyukkartal et al. (2005) considered that persistent tapetum blocked nutrient transportation and then led to low pollen fertility and affected the proportion of seed setting and grain formation. Therefore, abnormal tapetal cells caused anthers abortion, which is a common phenomenon in plants. In the study, the anther began to abort at stage FI3 in *B. oldhamii* ‘Xia Zao’, accompanied by unsuccessful meiosis of microspore mother cells and abnormal development of the tapetum, resulting in pollen abortion. Even when the 2-cell pollen grains were formed, it could also be seen that microspores and tapetal cells degenerated together in *B. oldhamii* ‘Xia Zao’. This phenomenon provides a clue that the aberrant development of meiosis, tapetal cells, and microspores is associated with low seed setting rate in bamboo plants.

In this study, we identified a group of genes involved in meiosis, megasporogenesis, and tapetum development, which could be responsible for the abortion phenomenon during meiosis and pollen maturation. For example, a number of known genes involved in meiosis (**Supplementary Table S2**) were also identified in the study, such as TFs *AtTGA10* (orthogrouop 4636) and *AtSCC2* (orthogroup 5234), genes encoding mismatch repair proteins, *AtMSH4* (orthogroup 5792) and *AtRAD50* (orthogroup 9771), a meiotic specific protein *AtZIP4* (orthogroup 9504), and a gene in structure maintenance of chromosome *AtMSC4* (orthogroup 4970). The fact that many of these meiotic genes maintain high sequence conservation suggests they play a similar functional role in bamboo plants. Significantly, *AMS* is identified as a key gene involved in meiosis. *AMS* encodes a basic helix-loop-helix (*bHLH*) TF, which plays a pivotal role in tapetum development and microspore formation. It was reported that mutations in this gene can lead to tapetum abnormalities and microspore degeneration (Sorensen et al., 2003). For instance, *AMS* mutations downregulated the expression of the genes involved in meiosis in *Arabidopsis*, and many genes related to tapetum metabolism were also directly regulated by *AMS*. In addition, *AMS* may change mitochondrial permeability and induce tapetum PCD by inducing the expression of *LTP* family genes. For example, rice *TdR*, an *AMS* homologous gene, can directly regulate cysteine proteases related to PCD (Na et al., 2006). In this study, the *AMS* gene from *B. oldhamii* ‘Xia Zao’ has a high sequence similarity with that from rice (**Figure 6**). However, the down-regulation of *AMS* in flowering stage of *B. oldhamii* ‘Xia Zao’ (**Figure 6**) is different from the non-reduced expression of *AMS* in rice (**Figure 6**), suggesting that a noncanonical gene regulation might occur in the late stage of meiosis.

Previous studies have shown that MUTL proteins are involved in DNA mismatch repair and play an important role in maintaining the stability of the meiotic genome (Kolodner and Marsischky, 1999; Hoffmann and Borts, 2004). Low seed setting rate is a widespread characteristic of *MutL* homologues mutants in eukaryotes. Evidence from *Arabidopsis* confirm that the deletion of *AtMLH1* leads to a significant reduction in pollen collapse and fruit set (Dion et al., 2007). Jackson et al. (2006) also reported that two base insertions from mutant alleles of *AtMLH3* resulted in truncation of the *AtMLH3* protein, leading to significant decreases in fruit set in *Arabidopsis*. Kim et al. (2015) reported that *AcPMS1* may play a restorative role in CMS onion. In yeast, *PMS1*, *MLH1*, *MLH2*, and *MLH3* have been reported to be MUTL protein homologs, with *PMS1* and *MLH1* forming the major dimer involved in the repair of single mismatched bases (Bray and West, 2005). In this study, our comparative sequencing analysis revealed high sequence similarity of *PMS* CDS between *B. oldhamii* ‘Xia Zao’ and rice (**Figure 7A**). However, the insertion of eight bases in exon 3 of *PMS* in *B. oldhamii* ‘Xia Zao’ caused a shift mutation that resulted in a scrambled frame and led to a premature stop codon at alignment position 606 (**Figure 7A**). Therefore, we speculate that the premature termination of *PMS1* contributed to the failure of meiosis in *B. oldhamii* ‘Xia Zao’.

In addition to *PMS*, we are concerned that another gene that plays a major role in the regulation of pollen wall formation, *NEF1*, also has a nonsense mutation in *B. oldhamii* ‘Xia Zao’ (**Figure 7B**). Pollen exine is an essential component of male gametophyte protection, defense, and female gametophyte recognition *NEF1* encodes a membrane protein present in the plastids of the tapetum that may be involved in the transport of polysaccharide required for the synthesis of the primexine (Ariizumi et al., 2004; Wang., 2013). In *Arabidopsis*, *NEF1* was reported to have a significant role in regulating the formation of pollen exine, and the absence of this gene will prompt to a deficit in primary exine synthesis, sporopollen accumulation failures, and the formation of fragmented microspores (Ariizumi et al., 2004; Dong et al., 2005; Chang et al., 2012). Besides, *NEF1* has a high degree of evolutionary conservation, with many plant genomes containing a single copy of the *NEF1* homolog, and this conserved structure ensures similarity in gene function (Wang, 2013). Only a single copy of *NEF1* was found in *B. oldhamii* ‘Xia Zao’, and its sequence is highly homologous to rice (**Figure 7B**). However, the presence of a base insertion behavior in the CDS region of *NEF1* in *B. oldhamii* ‘Xia Zao’ that distinguishes it from the rice CDS may be responsible for the distortion and rupture of the outer pollen wall (**Figures 2H, I** and **Supplementary Figure S10**).

**Figure 9:**
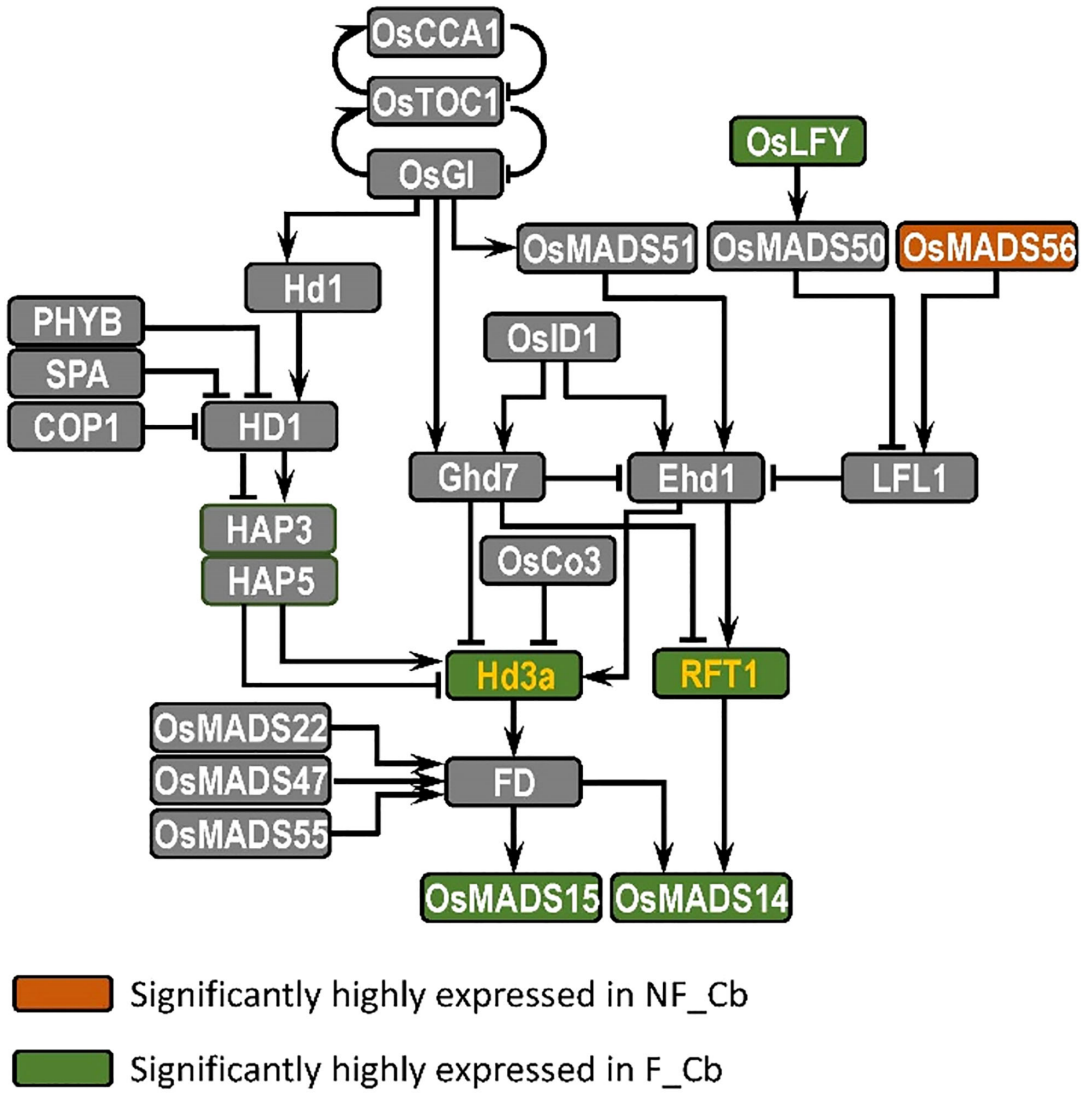
Mapping of DE genes in flowering and nonflowering culm buds in *B. oldhamii* ‘Xia Zao’ reveals the conserved evolution of photoperiod pathway. The yellow color represents integrated node gene.

Compared to other flowering plants, bamboo has a long nutritional growth and an irregular flowering period (Zheng et al., 2020), and we know little about the molecular mechanisms that regulate flowering time in bamboo. In this study, we have therefore analysed the transcriptome differences between nonflowering and flowering tissues of *B. oldhamii* ‘Xia Zao’ by high-throughput sequencing, leading to the identification of many genes involved in candidate flowering pathways in *B. oldhamii* ‘Xia Zao’. The evidence has revealed that only the genes involved in photoperiodic pathway are differentially expressed in the different tissues (**Supplementary Figure S9**). In *Arabidopsis*, three key genes *GIGANTEA* (*GI*), *CONSTANS* (*CO*) and *FT* in photoperiod pathway were reported to form a regulatory pathway to control photoperiod response and promote flowering in *Arabidopsis* (Suárez-López et al., 2001; Yanovsky and Kay, 2002; Putterill et al., 2004; Turck et al., 2008). There is a similar photoperiod pathway in rice, which is composed of *GIGANTEA* (*OsGI*), *Heading date 1* (*Hd1*) and *Heading date 3a* (*Hd3a*), which are homologous to *GI*, *CO* and *FT*, respectively (Yano et al., 2000; Shoko et al., 2002; Yang et al., 2015). In this study, we screened the photoperiod flowering network of *B. oldhamii* ‘Xia Zao’ and detected six photoperiod pathway node genes homologous to rice: *LEAFY*, *OsMADS56*, *Hd3a*/*RFT1* and *OsMADS14*/*OsMADS15* (**Figure 9**). Except for *OsMADS56*, the other five genes were highly expressed in flowering culm buds of *B. oldhamii* ‘Xia Zao’. *FT* gene acts as a key floral pathway integrator, essential for determining flowering time. It acts as an important flower-forming element that is transported to the SAM and thus induces flowering in plants (Corbesier et al., 2007; Tsuji et al., 2013). *Hd3a* and *RFT1*, which are highly homologous to *FT* as florigenic genes under short- and long-day conditions, respectively, are the main flowering inducers in rice (Shoko et al., 2002; Komiya et al., 2008). *OsMADS14* and *OsMADS15*, two genes *AP1*-like genes, located downstream of flower-forming elements, have been shown to be positive regulators of flowering in rice (Kobayashi et al., 2012). *LEAFY*, a transcriptional regulator that determines the formation of floral meristem, is highly conserved in different plant species (Weigel et al., 1992). Most known *LEAFY* genes are expressed in the vegetative and floral meristem (Weigel et al., 1992; Carmona, 2002; Wang et al., 2012; Yang et al., 2017; Hu et al., 2020). For example, homologous gene of *LEAFY*, *RFL* was expressed in only young panicles in rice (Kyozuka et al., 1998). *LiLFY1* was expressed in only young flower buds and apical meristems in lily (Wang et al., 2008). In this study, we assembled the full-length coding sequences of *FT* and *LFY* using the genome of *B. oldhamii* ‘Xia Zao’, which show a high degree of conservation with rice. However, the expression pattern of the genes displays a completely opposite trend in the two plant species. This suggests a degree of mis regulation of flowering-related genes may be related to the peculiar flowering characteristics of bamboo plants.

## Conclusion

Bamboo species represents a good evolutionary model to study flowering evolution, due to its predominant asexual reproduction using clonal propagation. We thus hypothesize that its flowering pathway must have relaxed to show non-conserved sequence evolution. This signature identified a cluster of genes including *FT* and *AMS*, two known key regulators of flowering and meiotic development. We examined in great detail of the sequence and expression evolution of these two genes, as well as another floral regulator, *LEAFY*, by using the genome of moso bamboo and conserved sequences in rice. Surprisingly, instead of relaxed sequence constraint, the full-length coding sequence of these three genes all retain high level of sequence conservation. *LEAFY* also shows its induced expression in flowering culm buds compared to the culm buds of nonflowering bamboo, indicating the presence of a functional flower and meiotic pathway for at least these three genes. In addition, by comparing to the documented transcriptional profile in rice, all three genes show an opposite expression trend. These lines of evidence indicate that gene regulation instead of protein sequence evolution may underlie the reduced flowering and meiotic failure in *B. oldhamii* ‘Xia Zao’. In addition to these master regulators, our analyses of sequence evolution revealed quite conserved sequence evolution for additional meiotic genes including TFs (*AtTAG10*, *AtZIP4*) and genes involved in mismatch repair (*AtRAD50*, *AtMSH4*). However, two genes – *PMS1* and *NEF1* show INDEL structural variation that disrupt the frame, and the high level of sequence conservation in the CDS with rice suggests that they are rather recent events. The processes these two genes contribute to and the associated meiotic abortion indicate a potential link between them and reduced seed production in *B. oldhamii* ‘Xia Zao’.

## Data availability statement

The data presented in the study are deposited in the NCBI SRA repository, accession number PRJNA876039.

## Author contributions

SL designed the research. WZ, LZ, and WY carried out the experiment. ZY and CG performed the bioinformatics analyses of the transcriptome data. WZ, CG, and WY wrote the manuscript. ZY and YD revised the manuscript. All authors contributed to the article and approved the submitted version.

## Funding

This work was financially supported by the National Key Research & Development Program of China (2021YFD2200503), the National Natural Science Foundation of China (Grant No. 31870595; No. 32001292) and the Priority Academic Program Development of Jiangsu Higher Education Institutions.

## Acknowledgments

The authors thank Muthusamy Ramakrishnan from Nanjing Forestry University for critical reading and editing of the manuscript. We also thank Mr. Shixing Zhang and Ruji Zhu at Xiapu bamboo breeding base for providing us with information of bamboo floral materials.

## Conflict of interest

The authors declare that the research was conducted in the absence of any commercial or financial relationships that could be construed as a potential conflict of interest.

The raw data and some figures used in the present study can be found in the electric supplementary material.

## Publisher’s note

All claims expressed in this article are solely those of the authors and do not necessarily represent those of their affiliated organizations, or those of the publisher, the editors and the reviewers. Any product that may be evaluated in this article, or claim that may be made by its manufacturer, is not guaranteed or endorsed by the publisher.
