# Supplementary figures and images for: Comparative phylogenomic analyses and co-expression gene network reveal insights in flowering time and aborted meiosis in woody bamboo, Bambusa oldhamii ‘Xia Zao’ ZSX
Source: Front Plant Sci. 2022 Nov 9;13:1023240. doi: 10.3389/fpls.2022.1023240 (PMC9681927; doi:10.3389/fpls.2022.1023240)

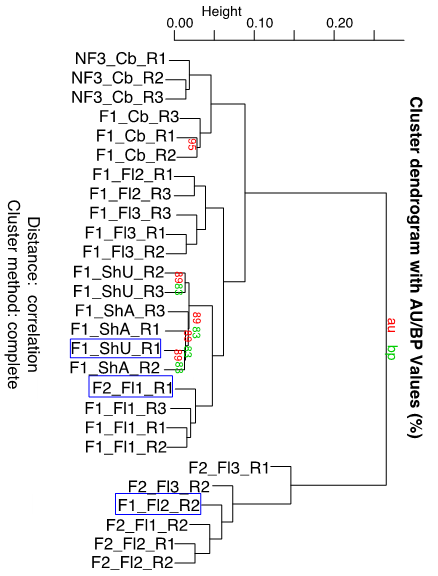

Supplement: Supplementary file 1 [file Image_1.tif]

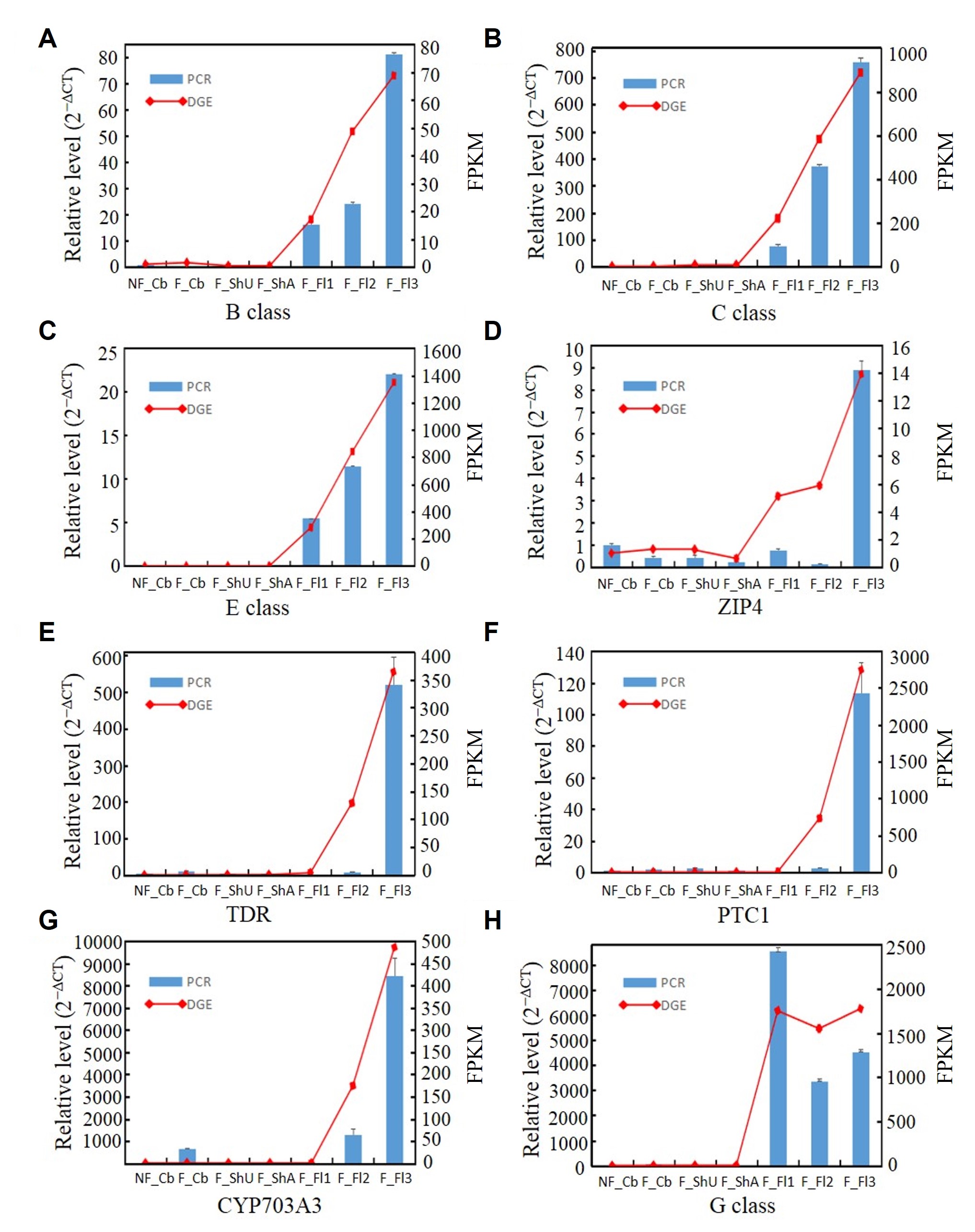

Supplement: Supplementary file 2 [file Image_2.jpg]

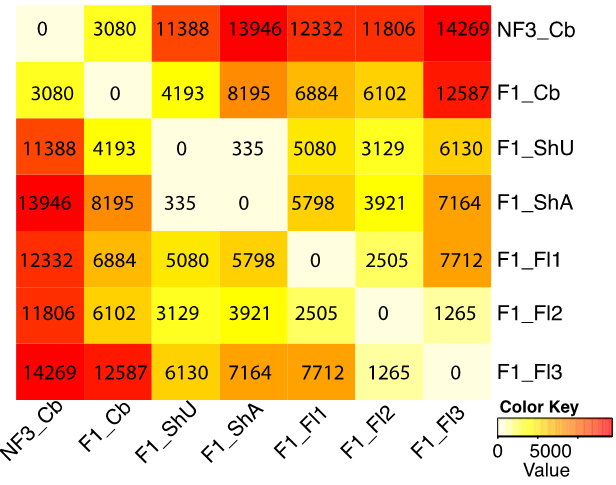

Supplement: Supplementary file 3 [file Image_3.tif]

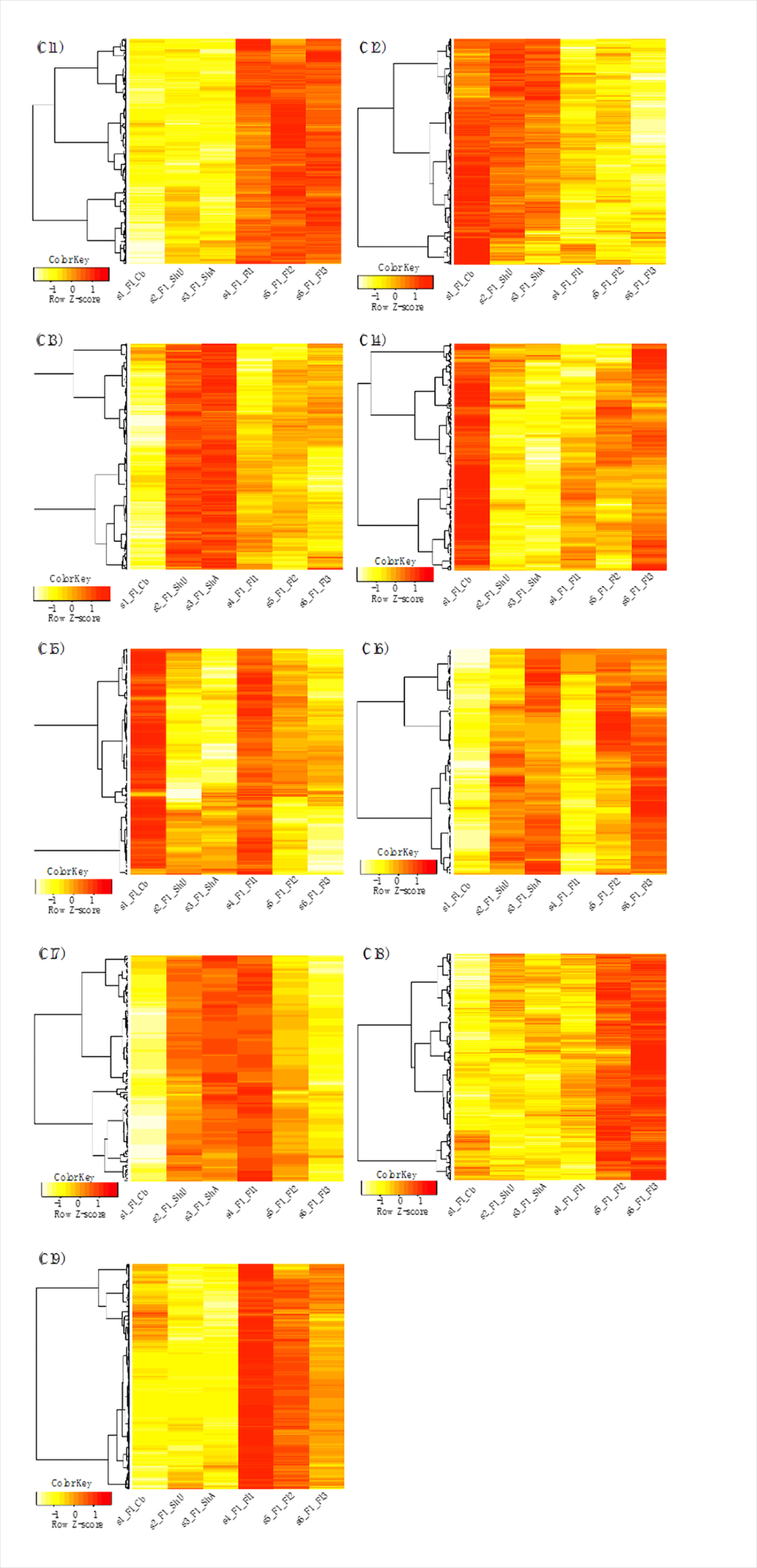

Supplement: Supplementary file 4 [file Image_4.tif]

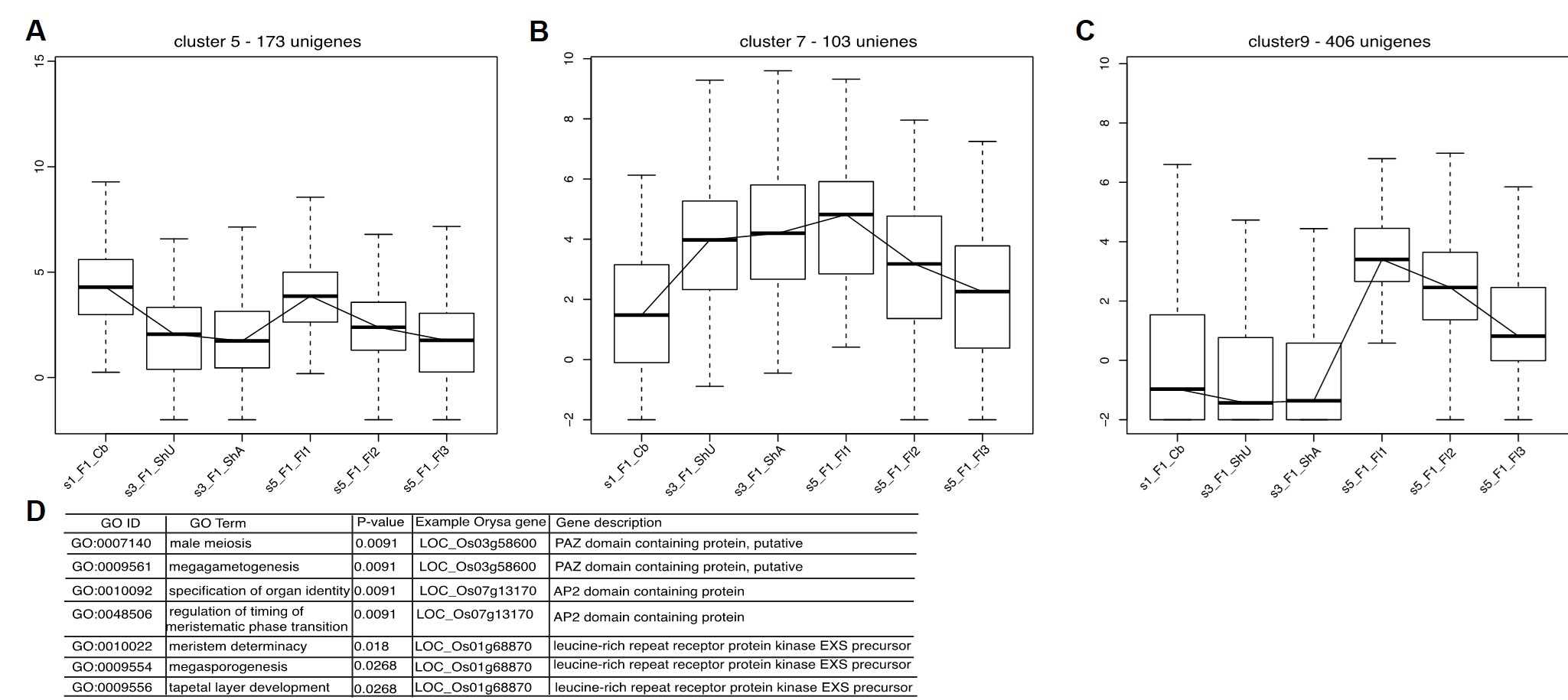

Supplement: Supplementary file 5 [file Image_5.jpg]

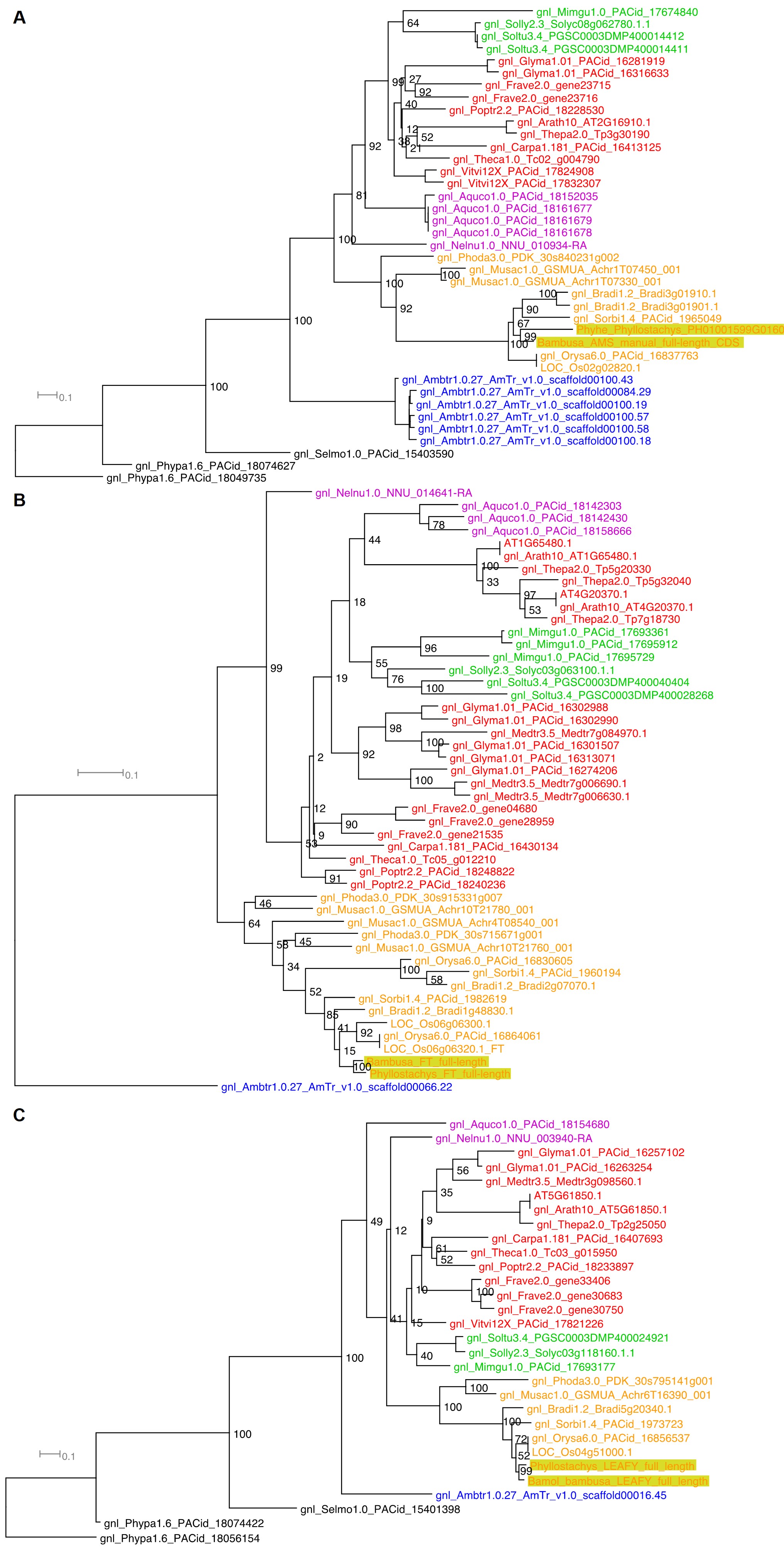

Supplement: Supplementary file 6 [file Image_6.jpg]

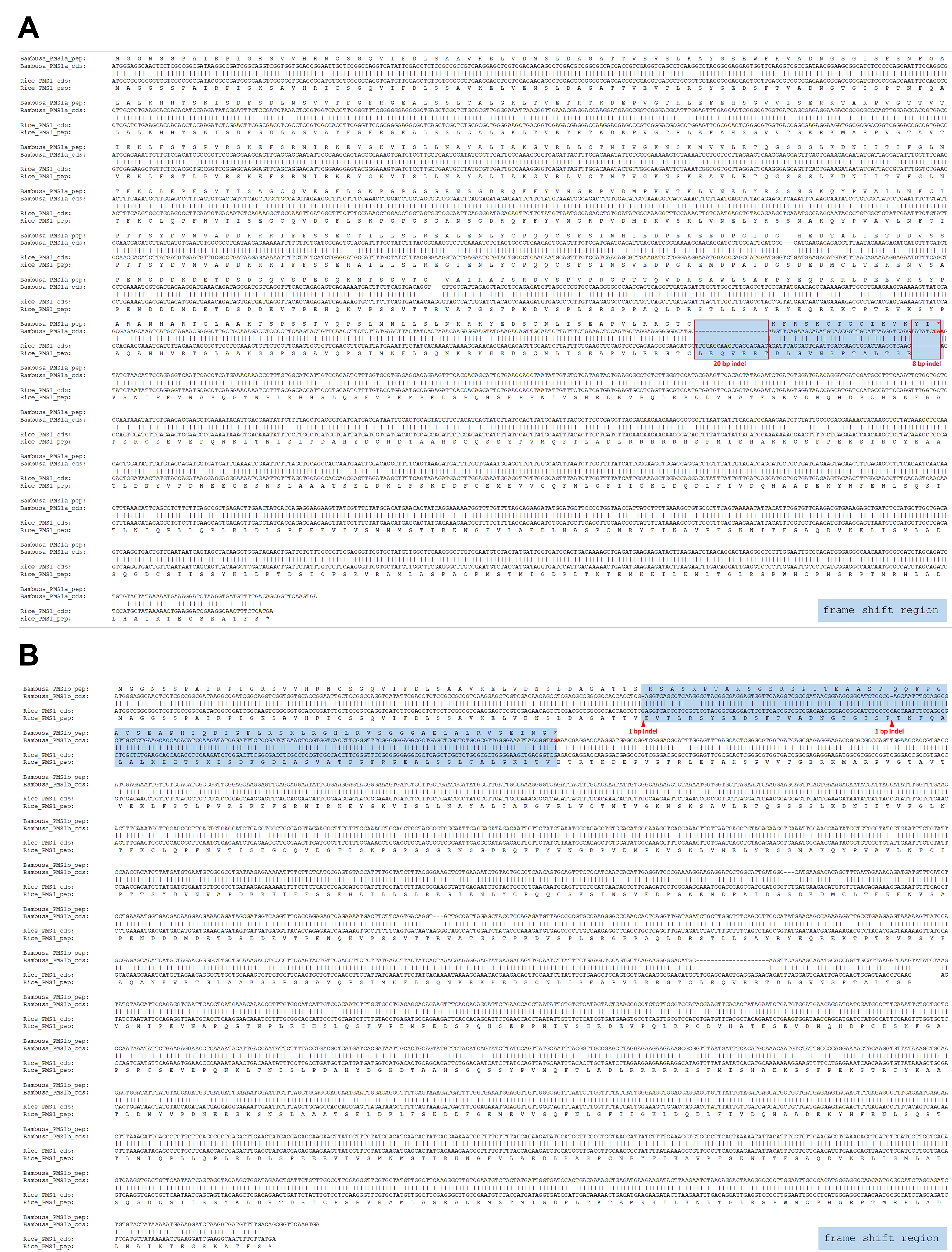

Supplement: Supplementary file 7 [file Image_7.tif]

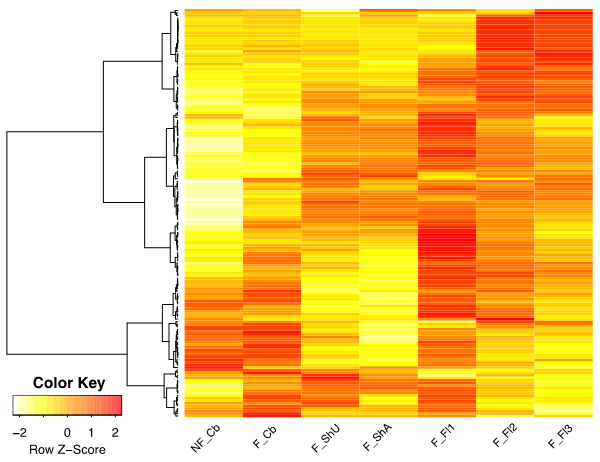

Supplement: Supplementary file 8 [file Image_8.tif]

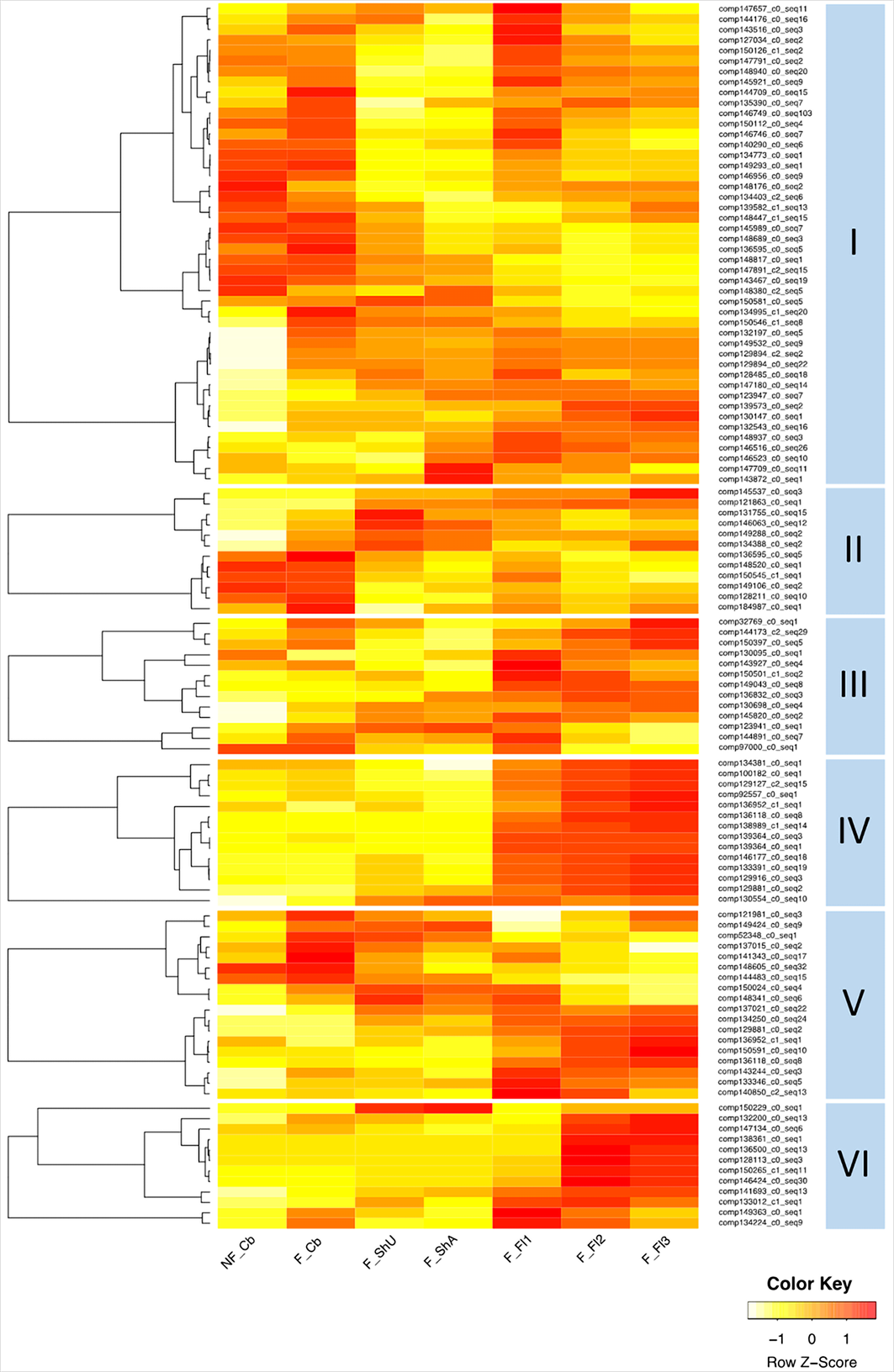

Supplement: Supplementary file 9 [file Image_9.tif]

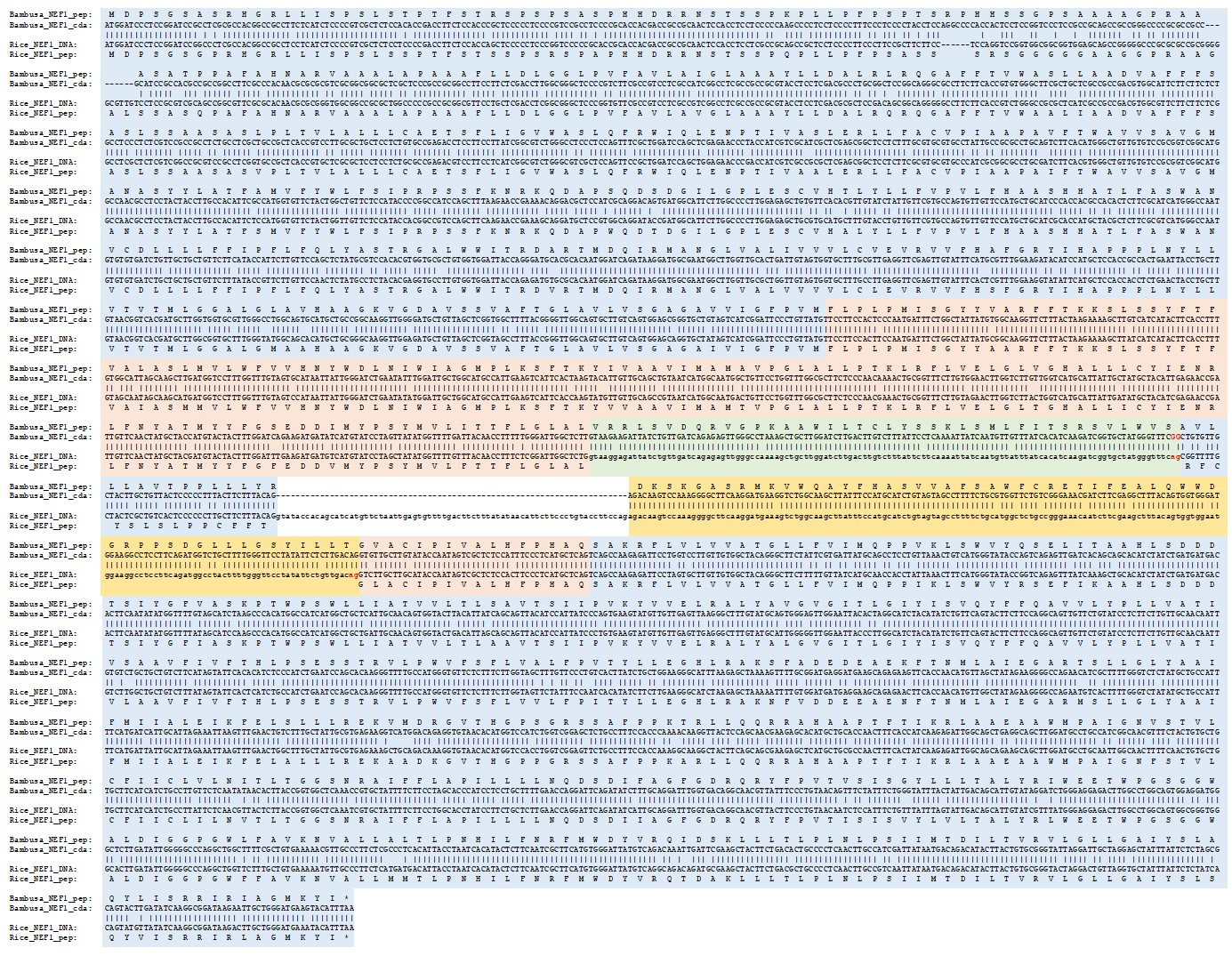

Supplement: Supplementary file 10 [file Image_10.jpeg]
